# Evaluation of two distinct placental‐derived membranes and their effect on tenocyte responses in vitro

**DOI:** 10.1002/term.2876

**Published:** 2019-06-13

**Authors:** John P. McQuilling, Kelly A. Kimmerling, Miranda C. Staples, Katie C. Mowry

**Affiliations:** ^1^ Research and Development Organogenesis Birmingham Alabama; ^2^ Milestone Research Organization San Diego California

**Keywords:** amniotic membrane, dehydrated amnion/chorion membrane (dACM), hypothermically stored amniotic membrane (HSAM), regenerative healing, tendon repair

## Abstract

Tendon healing is a complex, multiphase process that results in increased scar tissue formation, leading to weaker tendons. The purpose of this study was to evaluate the response of tenocytes to both hypothermically stored amniotic membrane (HSAM) and dehydrated amnion/chorion membrane (dACM). Composition and growth factor release from HSAM and dACM were evaluated using proteomics microarrays. HSAM and dACM releasate was used to assess tenocyte proliferation, migration, gene expression, extracellular matrix (ECM) protein deposition, and response to inflammation. Additionally, tenocyte–ECM interactions were evaluated. HSAM and dACM contain and release growth factors relevant to tendon healing, including insulin‐like growth factor I, platelet‐derived growth factor, and basic fibroblast growth factor. Both dACM and HSAM promoted increased tenocyte proliferation and migration; tenocytes treated with dACM proliferated more robustly, whereas treatment with HSAM resulted in higher migration. Both dACM and HSAM resulted in altered ECM gene expression; dACM grafts alone resulted in increases in collagen deposition. Furthermore, both allografts resulted in altered tenocyte responses to inflammation with reduced transforming growth factor beta levels. Additionally, dACM treatment resulted in increased expression and production of matrix metalloprotease‐1 (MMP‐1), whereas HSAM treatment resulted in decreased production of MMP‐1. Tenocytes migrated into and remodeled HSAM only. These results indicate that both grafts have properties that support tendon healing; however, the results presented here suggest that the responses to each type of graft may be different. Due to the complex environment during tendon repair, additional work is needed to evaluate these effects using in vivo models.

## 1 INTRODUCTION

In the United States, there are approximately 32 million musculoskeletal injuries annually; of these, approximately 45% are tendon and ligament injuries (Yang, Rothrauff, & Tuan, 2013); 300,000 of the 14.4 million patients suffering a tendon or ligament injury will undergo surgical repair (Yang et al., 2013). Although surgical repair is generally successful in restoring tendon function, adequate healing may be a concern, particularly for patients with multiple comorbidities (Ackermann & Hart, 2016). In addition to adequate tendon healing, another concern is the formation of adhesions to surrounding tissue caused in part by interruption of the synovial sheath (Matthews & Richards, 1976). Adhesion formation disrupts the normal gliding mechanics and consequently results in severe limitations in the functionality of the affected tendon. For flexor tendon repair, the occurrence of adhesion formation has been estimated at 4–10%, representing a significant number of adverse outcomes following surgery (Dy, Hernandez‐Soria, Ma, Roberts, & Daluiski, 2012; Tang, 2005).

Currently, many clinicians employ the use of cellular or acellular grafts to reinforce tendon repair sites with the goal of improving healing outcomes and reducing adhesion formation. One class of grafts available is human placental tissue grafts (Riboh, Saltzman, Yanke, & Cole, 2016). Although these grafts do not provide mechanical strength for physically reinforcing the repair, they contain a multitude of factors that are potentially beneficial to repair as indicated by recent studies. In vivo transplanting amniotic cells in a sheep tendinopathy model has shown improved mechanical and structural repair and faster ECM remodeling into mature collagen (Barboni et al., 2012). Additionally, a rodent study treating tendon lesions with amniotic membrane resulted in reduced healing times, improved collagen fiber alignment, a reduction in the inflammatory response, and an increase in tenoblast proliferation (Nicodemo et al., 2017). In limited early clinical work, patients receiving treatment with a liquid form of placental tissue matrix reported reduced pain and improved function of the treated appendage (Lullove, 2015; Warner & Lasyone, 2014; Werber, 2015). Although this existing research has demonstrated the potential for placental tissue to support tendon repair, few studies have utilized commercially available grafts.

The purpose of this study was to evaluate the effects of two commercially available placental‐derived membranes on tenocyte responses in vitro. We hypothesized that placental‐derived grafts would interact with tenocytes in vitro in ways that are expected to be beneficial to tendon repair. Second, we hypothesized that the processing methodologies and components of these grafts would influence how placental membranes interact with tenocytes in vitro. The two grafts evaluated in this study included a viable amnion‐only graft and a nonviable dehydrated graft consisting of both amnion and chorion membranes. The viable amnion‐only graft, also referred to as a hypothermically stored amniotic membrane (HSAM), contains amniotic membrane maintained in a proprietary solution at 1–10°C in order to preserve the cellular viability and overall integrity of the membrane. Growth factors, cytokines, and mechanisms by which HSAM may support wound healing responses in vitro have been previously evaluated (McQuilling, Vines, & Mowry, 2017). Evidence from McQuilling, Vines, and Mowry (2017) and others (Cooke et al., 2014; Niknejad et al., 2008) suggest that the preservation of placental ECM results in a scaffolding structure that new tissue can be built upon. By contrast, the nonviable dehydrated amnion/chorion membrane (dACM) contains both the amniotic membrane and the adjacent chorion membrane. In addition to the growth factors present in the amniotic membrane, the inclusion of the thicker chorion has been shown to increase the amount of growth factors present within the graft, increase allograft shelf‐life, and increase dwell time in vivo, despite the loss of some growth factors due to dehydration (McQuilling, Vines, Kimmerling, & Mowry, 2017).

## 2 METHODS

### 2.1 Human tissue allografts

HSAM (Affinity®, Organogenesis) and dACM (NuShield®, Organogenesis) are human allografts derived from placental tissue. Placentas were donated with informed consent after planned cesarean sections, and all processing was completed in accordance with the Food and Drug Administration's (FDA) Good Tissue Practices and the American Association of Tissue Banks Standards. HSAM is an aseptically processed HSAM stored in a proprietary storage solution, Allofresh™. dACM is a dehydrated amnion/chorion membrane that is terminally sterilized and stored at room temperature. All placental tissue donations underwent medical social history screening and were tested for infectious diseases including human immunodeficiency virus, human T‐lymphotropic virus I/II, hepatitis B and C, and syphilis.

### 2.2 Proteomic analysis of dACM and HSAM

To determine the concentrations of specific growth factors, 1‐cm^2^ samples from both HSAM (nine human tissue donors) and dACM (seven human tissue donors) were assessed utilizing a quantitative multiplex enzyme‐linked immunosorbent assay (ELISA) proteomics microarray. Growth factors selected for this analysis were (a) previously identified at physiological levels within amniotic membranes and (b) identified to be relevant to tendon healing pathways. These growth factors included insulin‐like growth factor I (IGF‐I), acidic and basic fibroblast growth factors (aFGF and bFGF), platelet‐derived growth factor‐BB (PDGF‐BB), and transforming growth factor beta‐1 (TGF‐β1; Molloy, Wang, & Murrell, 2003; Steed et al., 2008).

Growth factors released from either HSAM or dACM were evaluated by measuring the content of growth factors (described above) within the conditioned media (CM). Briefly, CM were generated from separate donors for HSAM (*n* = 2 human donors) and dACM (*n* = 2 human donors) evaluated individually in duplicate. Grafts were incubated in a serum‐free media (Dulbecco's modified Eagle's medium solution with 0.1% human serum albumin [Cellastim, InVitria, Junction City, KS]) for 5 days at 4°C with continuous gentle rocking. CM were made at a concentration of 1 ml of serum‐free media per 1 cm^2^ of HSAM or dACM used. After 5 days of incubation, HSAM or dACM was removed from the media, and CM were filtered using a 0.22‐μm syringe filter. CM were then stored at 4°C until use (up to 14 days later).

### 2.3 Tenocyte isolation and culture

Commercially available tenocytes were obtained from Zen‐Bio (Durham, NC) from three male donors ages 61–81. Before use, tenocytes were confirmed positive for cell surface markers CD44 and CD90 and negative for CD45 and CD31 by the supplier. For our experiments, cells were passaged continuously (never frozen), and cells from passages 3 and 4 were used. For all experiments, seeding densities were determined on the basis of manufacturers' recommendations, where appropriate or otherwise determined using pilot experiments.

### 2.4 Tenocyte proliferation assay

Cell proliferation assays were conducted with adult human tenocytes as described above. Tenocytes were seeded into 48‐well plates at a concentration of 3,300 cells per well and cultured in the presence or absence of CM from HSAM or dACM. To evaluate cell proliferation, AlamarBlue (Invitrogen, Carlsbad, CA) assays were conducted according to manufacturer's instructions. Briefly, at each time point evaluated (1, 4, 7, and 10 days), media in each well was aspirated; cells were then incubated in 350 μl of a 10% AlamarBlue working solution in assay media (Dulbecco's modified Eagle's medium with 2.5% fetal bovine serum, antibiotics, and l‐glutamine) for 4 hr under standard culture conditions. Following incubation with AlamarBlue, fluorescence was measured in duplicate using a plate reader (Synergy H1, BioTek, Winooski, VT) from 100 μl of samples using a fluorescence excitation wavelength of 540–570 nm and fluorescence emission wavelength of 580–610 nm. Immediately following evaluation, AlamarBlue solution was replaced with appropriate media for each condition. Cell number was determined using standards created with tenocytes plated at known concentrations.

### 2.5 Tenocyte migration assays

The effect of HSAM or dACM on cell migration was assessed with a standard Boyden chamber assay as previously described in detail (McQuilling, Vines, & Mowry, 2017). Briefly, tenocytes were added to the top of the chamber at a concentration of 5,000 cells per insert and then incubated for 24 hr to allow for cell migration. Cell migration was measured in response to serum‐free media (negative control), assay media + 10% fetal bovine serum (positive control), or CM from either HSAM or dACM at concentrations of 50%, 25%, and 10% (v/v). Images of the inserts were taken with an inverted microscope (Nikon Eclipse Ti, Tokyo, Japan), and migrated cells were counted.

Alternatively, tenocyte migration was evaluated using a standard scratch assay (Liang, Park, & Guan, 2007). Tenocytes were seeded into 24‐well plates at a concentration of 25,000 cells per well and cultured overnight. Monolayers of tenocytes were scratched with a P200 pipette tip. Tenocytes were then cultured with either assay media alone (control) or with assay media containing 25% CM (v/v) from either HSAM or dACM. Preselected regions of the scratched monolayer were then imaged with an inverted microscope (Nikon Eclipse Ti, Tokyo, Japan), at 0, 4, 8, 16, and 24 hr postinjury. Total scratch area was then measured using Image J software (NIH, Bethesda, MD), and percent closure was determined at each time point on the basis of initial scratch area.

### 2.6 Tenocyte responses to inflammatory cytokines

To evaluate the effects of placental‐derived membranes on tenocytes under inflammatory conditions, tenocytes were cultured in the presence of inflammatory cytokines [tumor necrosis factor alpha (TNF‐α) or interleukin 1 beta (IL‐1β)] with or without CM from HSAM or dACM. Briefly, 40,000 tenocytes were seeded per well into six‐well plates with growth media and cultured overnight under standard culture conditions. Tenocytes were then cultured in assay media alone or in assay media containing TNF‐α or IL‐1β (1 or 0.1 ng/ml) to simulate inflammatory conditions. To model treatment with placental‐derived membranes, 50% CM from either dACM or HSAM were added to half the wells. At the end of the 96‐hr culture period, supernatant was collected and stored at −80°C for use in ELISAs, and the cell content per well was quantified using AlamarBlue (Invitrogen, Carlsbad, CA) prior to collection for reverse transcription–polymerase chain reaction.

### 2.7 Gene expression and ELISAs

Tenocyte production of matrix metalloprotease‐1 (MMP‐1) and TGF‐β1 in response to inflammatory cytokines was measured via commercially available ELISA kits (Fisher Scientific, Waltham, MA) per manufacturer's instructions. For all gene expression experiments, cells were collected in RNAzol RT (Thermo Scientific, Waltham, MA) and stored at −80°C until analysis. Total RNA was extracted and quantified on a NanoDrop 2000 UV/Vis spectrophotometer (Thermo Scientific, Waltham, MA). Reverse transcription to cDNA was performed using the Verso cDNA synthesis kit (Thermo Fisher Scientific, Pittsburgh, PA). Levels of mRNA were measured using TaqMan probes and Fast Advanced Master Mix on a QuantStudio3 using a standard protocol (Applied Biosystems, Waltham, MA). Each experimental gene was normalized to glyceraldehyde 3‐phosphate dehydrogenase (*GAPDH*), and the relative expression of mRNAs was calculated by the 2^−ΔΔCt^ method. A complete list of the probes used is included in Table S1.

### 2.8 Gene expression and ELISAs

Tenocytes were seeded at 40,000 tenocytes per well into six‐well plates and cultured in assay media with or without 50% CM (dACM or HSAM) supplemented with 50 μM of ascorbic acid to allow for the deposition of collagen (Hakimi, Poulson, Thakkar, Yapp, & Carr, 2014). After 1 week of culture, the quantity of total collagen and sulfated glycosaminoglycans (sGAGs) was evaluated using the Sircol soluble collagen and Blyscan glycosaminoglycan assays, respectively (Biocolor, UK). Total ECM content was subsequently normalized to total DNA content, which was determined using a Quant‐iT™ PicoGreen™ dsDNA Assay Kit (ThermoFisher, Waltham MA). Additionally, gene expression for relevant targets, including ECM‐related genes, was evaluated in tenocytes cultured for 96 hr as detailed previously.

### 2.9 Tenocyte culture with dACM and HSAM

To qualitatively analyze the interaction of tenocytes with HSAM and dACM grafts, 50,000 tenocytes were seeded onto dACM or HSAM (2.5 cm^2^, *n* = 3 from the same donor with HSAM and dACM originating from two distinct donors). Of note, grafts without tenocytes seeded were cultured concurrently and used for comparison. Grafts were then cultured in six‐well plates under standard conditions and collected at 3, 7, 14, and 21 days for histological analysis.

### 2.10 Histology

Postculture, grafts were fixed in a 4% solution of paraformaldehyde in phosphate‐buffered saline for 24 hr. Samples were paraffin embedded, and 5‐μm‐thick serial sections were cut from tissue blocks. Care was taken to maintain the sectioning plane for all samples; however, dramatic remodeling of some of the grafts made precise orientation difficult. Sections were floated onto charged glass slides (SuperFrost Plus, Fisher Scientific, Pittsburgh, PA) and dried overnight at 60°C. Both dACM and HSAM sections were stained for hematoxylin and eosin (H&E). Upon evaluation of H&E staining, further staining of HSAM sections was completed (Masson's trichrome, Verhoeff's stain, and Alcian Blue).

Based on observations from H&E‐stained sections, tenocytes migrated into and begin to remodel HSAM grafts but only formed a monolayer along dACM grafts. To further assess remodeling observed with HSAM grafts, sections were also stained for immunohistochemical (IHC) evaluation of collagen I, collagen III, decorin, and tenomodulin. All sections for IHC were deparaffinized and hydrated using graded concentrations of ethanol to deionized water. Tissues sections were subjected to antigen retrieval using a 0.01‐M Tris–1‐mM EDTA (pH 9) at 70°C for 20 min. Following antigen retrieval, all sections were washed gently in deionized water and then transferred to a 0.05‐M Tris‐based solution in 0.15‐M NaCl with 0.1% v/v Triton X‐100 (pH 7.6, Tris‐buffered saline, 0.1% Tween 20 (TBST)). Endogenous peroxidase was blocked with 3% hydrogen peroxide for 20 min. To reduce further nonspecific background staining, slides were incubated with 3% normal goat serum for 30 min (Sigma‐Aldrich, St. Louis, MO) at room temperature. All slides then were incubated at 4°C overnight with appropriate antibodies (collagen I, ab34710, Abcam, 1:100; collagen III, ab7778, Abcam, 1:100; decorin, PIPA527370, Thermo Scientific, 1:100; tenomodulin, ABC305MI, EMD Millipore, 1:100). Negative controls were produced by eliminating the primary antibodies from the diluents. After being washed with TBST, sections were incubated with matching secondary antibodies conjugated with horseradish peroxidase. Diaminobenzidine (ScyTek Laboratories, Logan, UT) was used as the chromogen and hematoxylin (Richard‐Allen Scientific, Kalamazoo, MI) as the counterstain. All stained samples were visualized using an inverted microscope (Nikon Eclipse Ti, Tokyo, Japan Nikon, Melville, NY) with objective of 20× and 40×.

### 2.11 Statistical analysis

Statistical analysis for the proteomics assays was conducted using an unpaired *t* test to compare protein levels between HSAM and dACM. For all other quantitative assays, statistical analysis was conducted using a one‐way analysis of variance with a post hoc Bonferroni test where *p* < .05 was considered significant. Unless otherwise specified, throughout this manuscript, * denotes *p* < .05, ** denotes *p* < .01, *** denotes *p* < .001, and **** denotes *p* < .0001.

## 3 RESULTS

### 3.1 Proteomic analysis of dACM and HSAM

**Figure 1:**
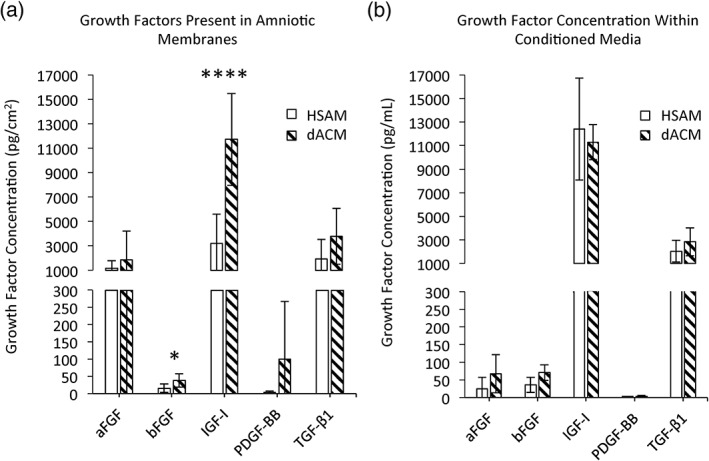
Evaluation of relevant tendon healing growth factors. (a) Growth factors present in amniotic membranes. Mean ± standard deviation reported; n = 9 for HSAM, n = 7 for dACM. * denotes p < .05; **** denotes p < .0001. (b) Growth factor concentration in conditioned media. Mean ± standard deviation reported; n = 4 for both HSAM and dACM. aFGF, acidic fibroblast growth factor; bFGF, basic fibroblast growth factor; dACM, dehydrated amnion/chorion membrane; HSAM, hypothermically stored amniotic membrane; IGF‐1, insulin‐like growth factor I; PDGF‐BB, platelet‐derived growth factor‐BB; TGF‐β1, transforming growth factor beta‐1

Proteomic microarrays confirmed physiologically relevant concentrations of growth factors related to tendon healing in both HSAM and dACM grafts (Figure 1a). Growth factors measured in this study include aFGF, bFGF, IGF‐I, PDGF‐BB, and TGF‐β1, which are all upregulated during the natural tendon repair process (Dahlgren, Mohammed, & Nixon, 2005; Evans, 1999). Some sample‐to‐sample variability is known to be present in placental tissues, and the data presented in this study fall within the observed ranges in previous studies of dehydrated amnion/chorion grafts and HSAM (Koob et al., 2013; Koob, Lim, Massee, Zabek, & Denozière, 2014; McQuilling, Vines, & Mowry, 2017). For overall quantities of growth factors per square centimeters, dACM grafts contained a significantly higher concentration of both bFGF (*p* < .05) and IGF‐I (*p* < .0001) than did the HSAM allograft, which is likely attributable to its dual layer (amnion/chorion; McQuilling, Vines, Kimmerling, & Mowry, 2017).

Additionally, we hypothesized that processing of dACM may affect growth factor release from the membranes due to the compression and increased density of the ECM caused by removing the moisture from the graft. To evaluate this, the quantities of aFGF, bFGF, IGF‐I, PDGF‐BB, and TGF‐β1 released from HSAM or dACM into assay media were quantified (Figure 1b). Interestingly, there were no significant differences in the quantity of released growth factors measured between dACM and HSAM grafts. Taken together, these results show that although dACM grafts contain higher overall growth factor loads, the quantities of growth factors released are comparable between HSAM and dACM. In sum, both grafts contain growth factors necessary to support tendon healing.

### 3.2 Tenocyte proliferation

**Figure 2:**
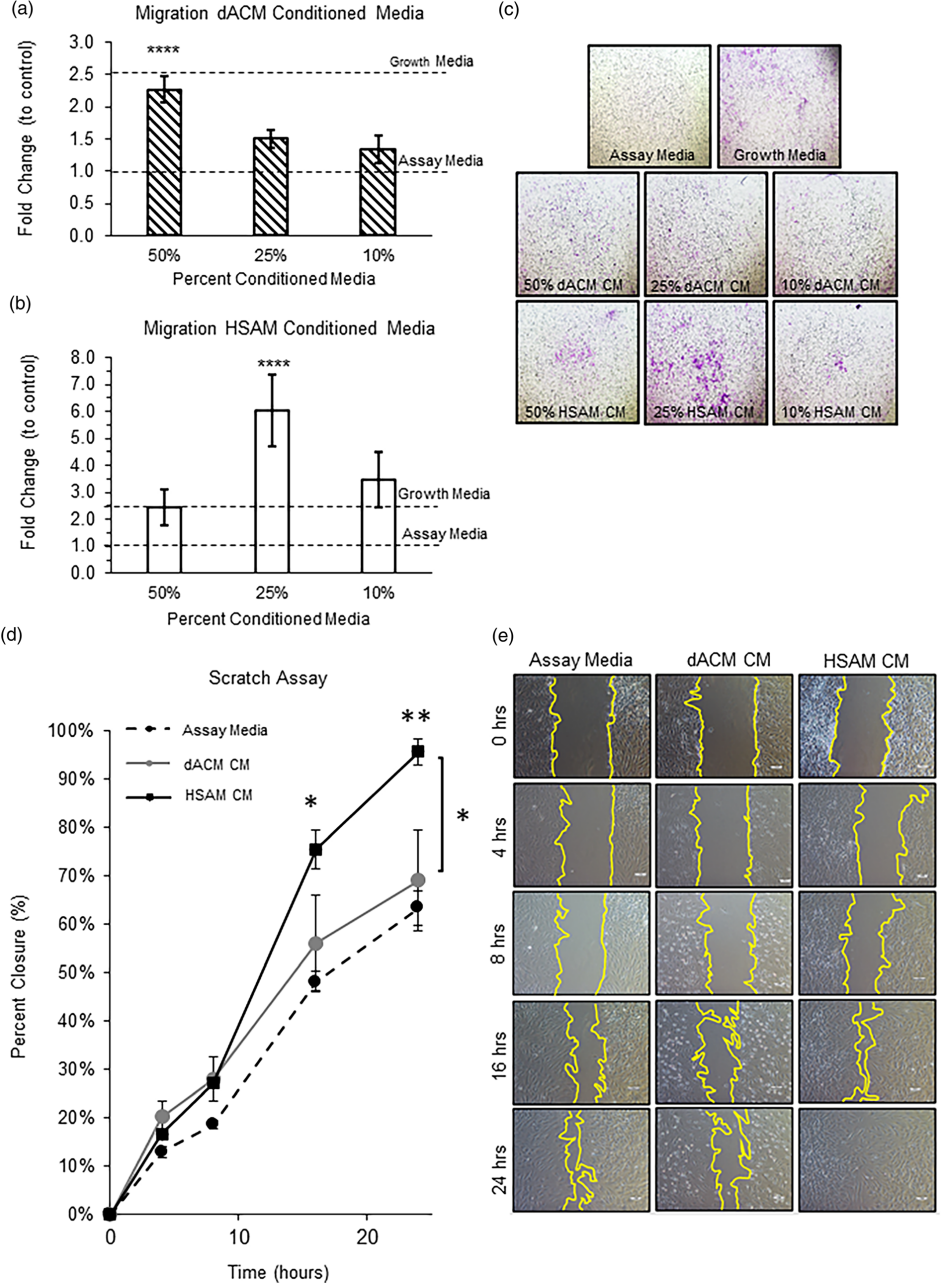
Tenocyte proliferation experiments. Total cell counts for (a) HSAM conditioned media (CM) and (b) dACM CM. Mean ± standard deviation reported; n = 12 for all groups. (c) Tenocyte proliferation at 14 days for both dACM and HSAM CM. Mean ± standard deviation reported; n = 12 for each group. ** denotes p < .01, and **** denotes p < .0001. (d) Representative images showing tenocyte proliferation for each group; 4× objective. dACM, dehydrated amnion/chorion membrane; HSAM, hypothermically stored amniotic membrane

In addition to studying specific growth factor concentrations and their release from the grafts, the effect of the released growth factors on important mechanisms of tendon healing was evaluated. Specifically, this included the effects of CM from either HSAM or dACM on tenocyte proliferation, migration, ECM deposition, and response to inflammatory stimulus. By Day 3, tenocyte proliferation was significantly upregulated in all CM groups following treatment with HSAM CM and dACM CM (*p* < .01) than in assay media controls (Figure 2a,b). This trend continued through Day 14 for dACM groups, where all three dilutions of CM continued to have significant increases in cell proliferation than did the assay media control (*p* < .0001). HSAM CM groups also continued to show significant increases in cell proliferation than did the assay media control to Day 14 (*p* < .01), with the exception of the 10% CM group at Day 7. When directly comparing dACM CM with HSAM CM at Day 14, CM from dACM resulted in a more robust increase in cell proliferation at all concentrations (*p* < .001, Figure 2c). Representative images illustrating tenocyte proliferation for each group are shown in Figure 2d. The observed increases in cell proliferation for dACM and HSAM groups are expected because CM contains growth factors known to promote tenocyte proliferation including IGF‐I, bFGF, and PDGF‐BB (Molloy et al., 2003; Nourissat, Berenbaum, & Duprez, 2015).

### 3.3 Tenocyte migration

**Figure 3:**
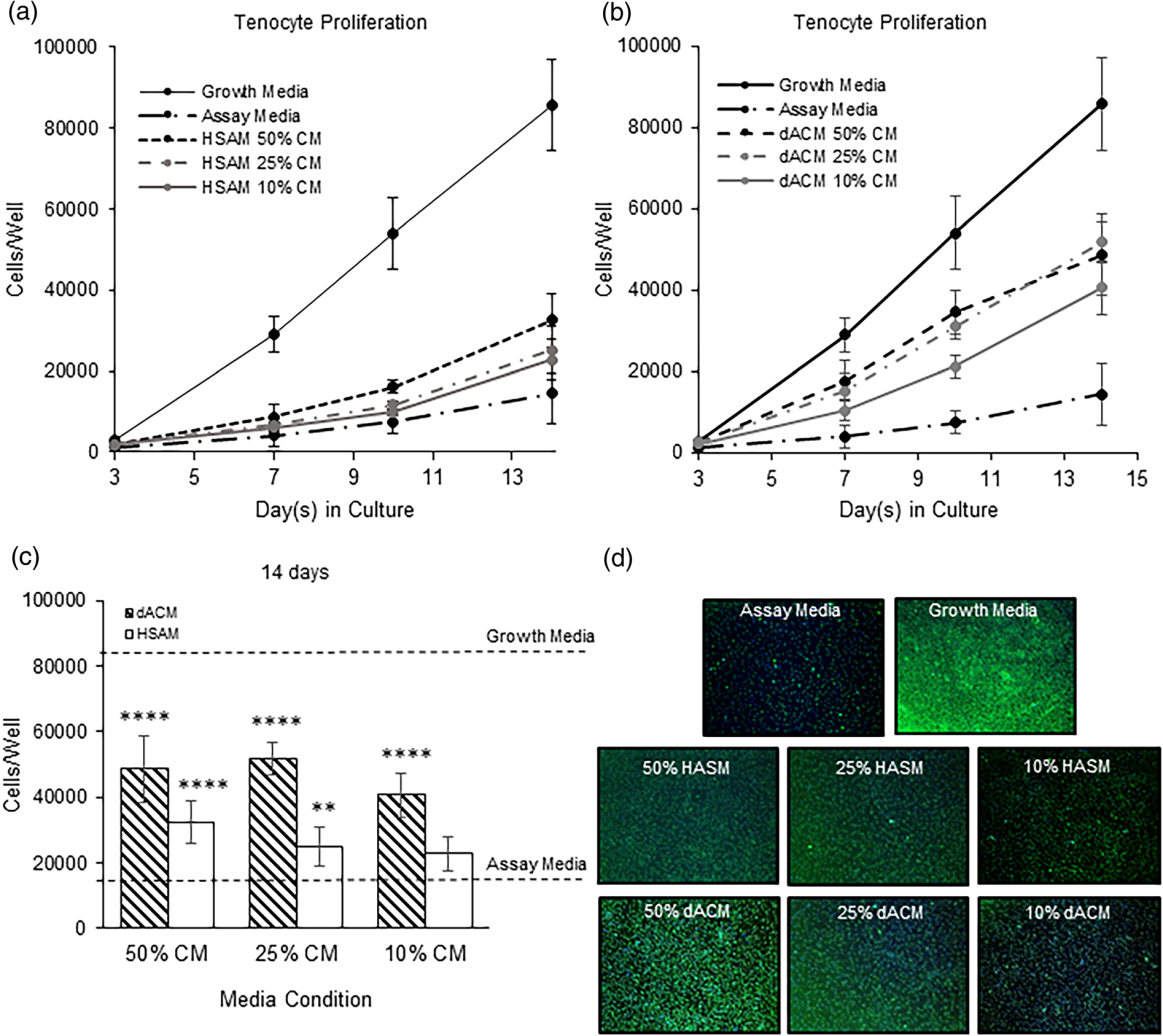
Tenocyte migration experiments. (a) Migration fold change for dACM conditioned media (CM) and (B) HSAM CM. Mean ± standard deviation reported; n = 24 for each group. **** denotes p < .0001 compared with assay media. (c) Representative images showing tenocyte migration for each group; 4× objective. (d) Scratch assay using assay media, dACM CM, and HSAM CM. Mean ± standard deviation reported; n = 6 for all groups. * denotes p < .05 compared with assay media or dACM CM; ** denotes p < .01 compared with assay media. (e) Representative images showing scratch assay closure for each group; scale bar indicates 100 μm. dACM, dehydrated amnion/chorion membrane; HSAM, hypothermically stored amniotic membrane

The effect of released growth factors on migration of tenocytes was evaluated by both Boyden chamber migration assays and standard scratch assays. With the use of Boyden chamber assays, both dACM and HSAM CM promoted tenocyte migration than did assay media. For the dACM CM migration experiments, migration of tenocytes was significantly increased with the 50% CM group compared with assay media (Figure 3a, *p* < .0001). For the HSAM CM migration experiments, tenocyte migration was significantly upregulated in the 25% CM group compared with assay media (Figure 3b, *p* < .0001). When comparing tenocyte migration between the groups, HSAM CM promoted more robust migration than did dACM CM in the 25% CM group (*p* < .01). Although the varying effects on migration observed here between HSAM and dACM warrant further investigation, this may be in part due to the biphasic nature of many chemoattractant molecules, where lower doses may be more effective at stimulating migration than are higher doses (Calabrese, 2001).

These results were further confirmed using a scratch assay, where a monolayer of tenocytes was scratched; repair of the scratch treated with assay media, dACM CM, or HSAM CM was quantified over 24 hr. At 16 and 24 hr, HSAM CM resulted in a significant increase in closure than did assay media (*p* < .05, *p* < .01; Figure 3d). Additionally, at 24 hr, releasate from HSAM resulted in significantly greater percent closure than did dACM CM (*p* < .05). Representative bright‐field images both illustrate percent closure and offer further explanation on how closure was measured (Figure 3e). Taken together with the findings of the Boyden chamber migration assay, CM from HSAM promoted more robust tenocyte migration than dACM CM. IGF‐I, TGF‐β, and FGF have been shown to support both proliferation and migration of tenocytes (Molloy et al., 2003).

### 3.4 Collagen deposition and ECM expression

**Figure 4:**
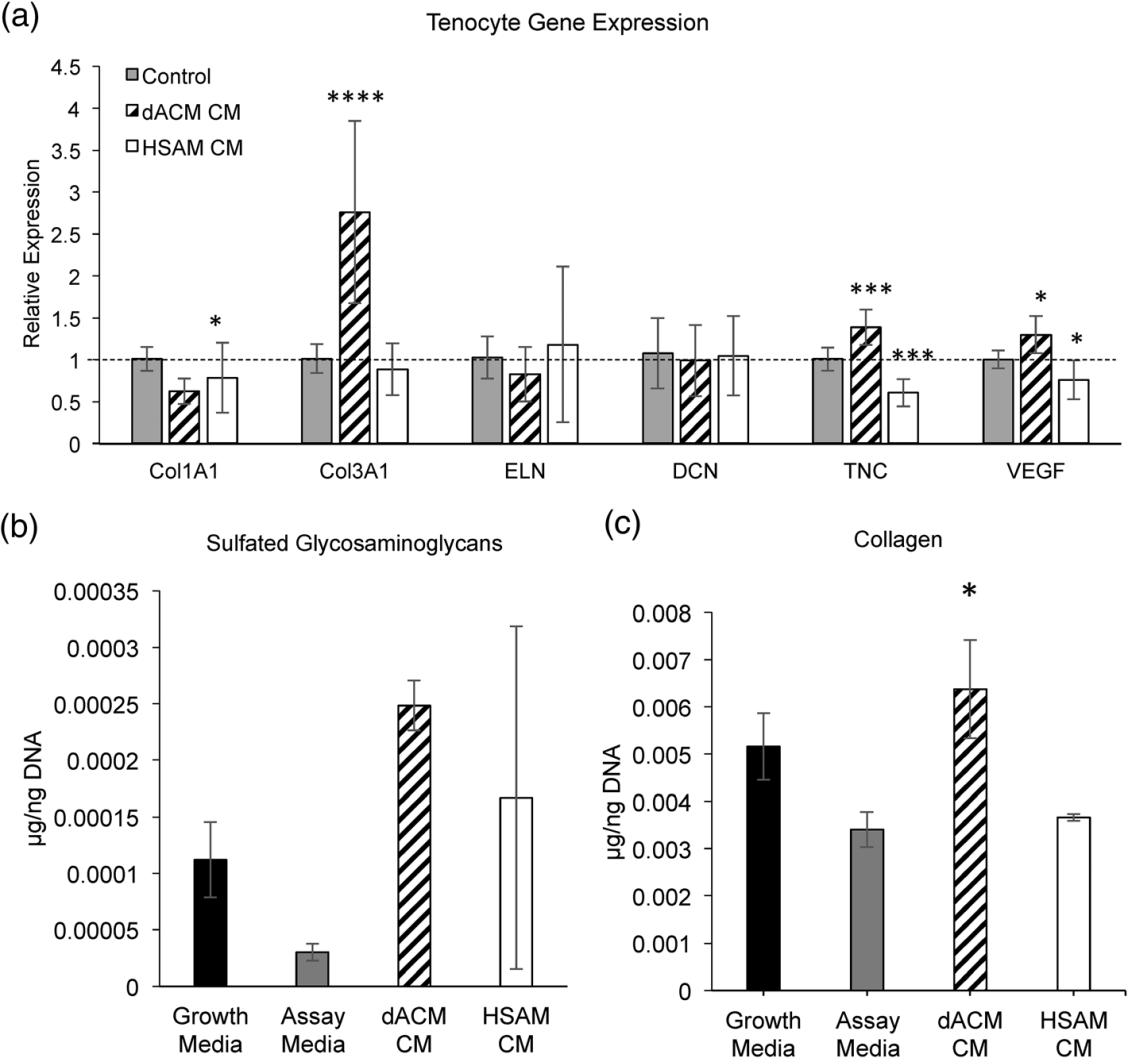
Tenocyte extracellular matrix gene expression and deposition. (a) Gene expression of collagen I (COL1A1), collagen III (COL3A1), elastin (ELN), decorin (DCN), tenascin C (TNC), and vascular endothelial growth factor (VEGF). Mean ± standard deviation reported; n = 9 for all groups. * denotes p < .05; *** denotes p < .001; **** denotes p < .0001 compared with control. (b) Deposition of sulfated glycosaminoglycans (sGAGs). Mean ± standard deviation reported; n = 3 for all groups. (c) Deposition of collagen. Mean ± standard deviation reported; n = 3 for all groups. * denotes p < .05 compared with assay media. CM, conditioned media; dACM, dehydrated amnion/chorion membrane; HSAM, hypothermically stored amniotic membrane

**Figure 5:**
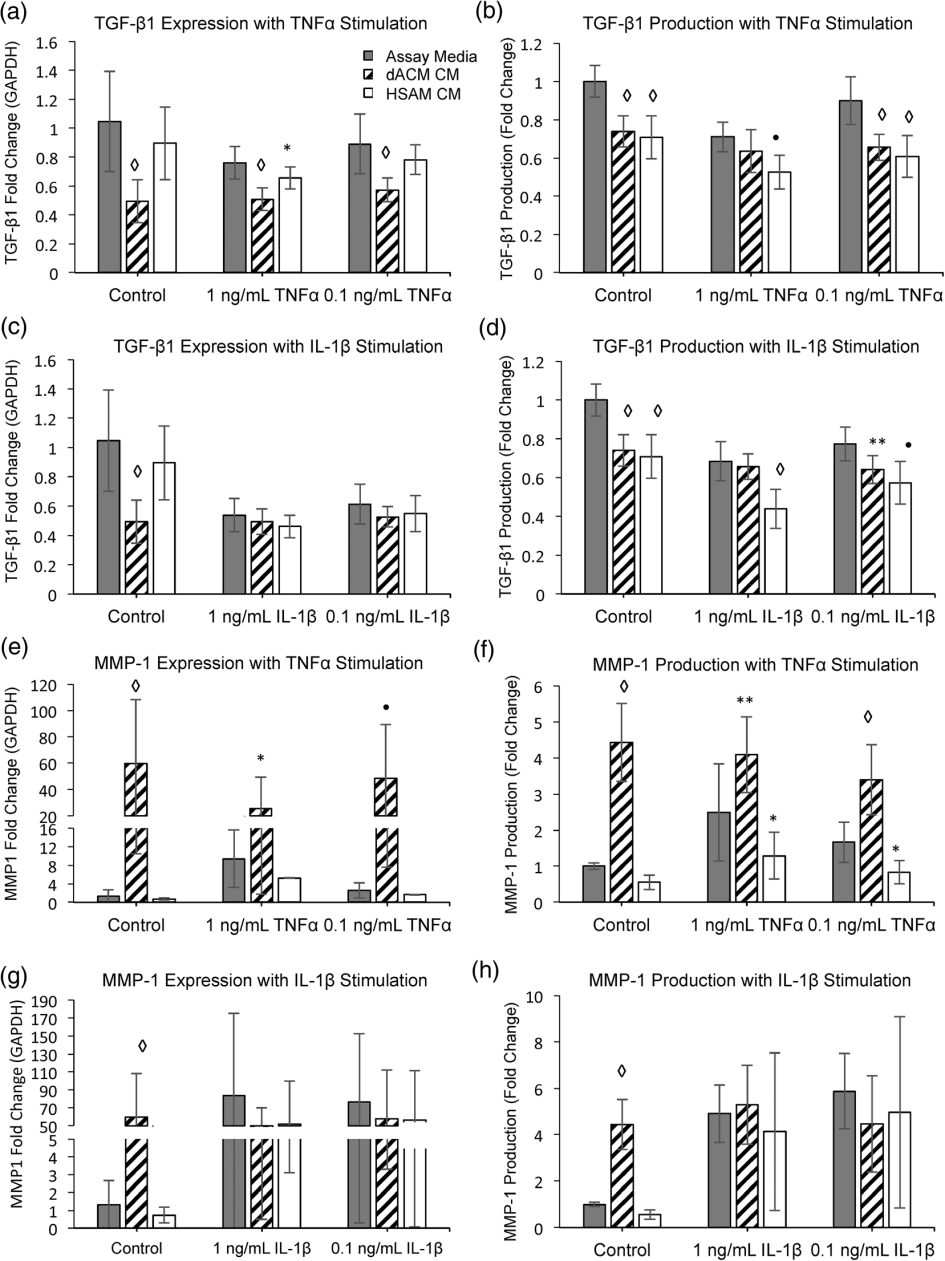
Tenocyte response to inflammatory cytokines. Gene expression fold change of TGFB1 after stimulation with (a) TNF‐α and (c) IL‐1β normalized to GAPDH. Mean ± standard deviation reported; n = 12 for all groups. * denotes p < .05; ● denotes p < .001; ◊ denotes p < .0001 compared with control. Protein production fold change of TGF‐β1 after stimulation with (b) TNF‐α and (d) IL‐1β normalized to assay media. Mean ± standard deviation reported; n = 9 for all groups. ** denotes p < .01; ● denotes p < .001; ◊ denotes p < .0001 compared with control. Gene expression fold change of MMP1 after stimulation with (e) TNF‐α and (G) IL‐1β normalized to GAPDH. Mean ± standard deviation reported; n = 12 for all groups. * denotes p < .05; ● denotes p < .001; ◊ denotes p < .0001. Protein production fold change of MMP‐1 after stimulation with (f) TNF‐α and (h) IL‐1β normalized to assay media. Mean ± standard deviation reported; n = 9 for all groups. * denotes p < .05; ** denotes p < .01; ◊ denotes p < .0001. CM, conditioned media; dACM, dehydrated amnion/chorion membrane; HSAM, hypothermically stored amniotic membrane; IL, interleukin; TGF‐β1, transforming growth factor beta‐1

Reconstruction of the ECM following tendon injury is a critical phase during the repair process. The early production of ECM components during the proliferative phase of healing is essential to quickly provide a functioning matrix over the defect area, whereas the later remodeling of this tissue is essential to restore the mechanical properties of the tendon. To evaluate the effect of dACM or HSAM on gene expression of targets related to tendon repair and ECM deposition, tenocytes were exposed to releasate from dACM or HSAM for 96 hr under standard culture conditions. Gene expression of targets important to tendon repair with dACM CM and HSAM CM‐treated tenocytes was performed (Figure 5a). The assessed genes include collagen 1A1 (*COL1A1*) and collagen 3A1 (*COL3A1*), which serve as precursors to collagen fibers and provide structure to the tendon (Voleti, Buckley, & Soslowsky, 2012); elastin (*ELN*), which provides elasticity to collagen fibers under tensile strain (Muiznieks & Keeley, 2013); decorin (*DCN*), which serves to bind collagen fibrils together, particularly type I and type IV collagen fibrils (Seidler & Dreier, 2008); tenascin (*TNC*), which serves to facilitate cellular migration through the ECM (Chiquet‐Ehrismann, 2004); and vascular endothelial growth factor (*VEGF*), which serves as a potent stimulator of angiogenesis during tendon repair (Yang et al., 2013). In dACM CM‐treated tenocytes, *COL1A1* was significantly decreased (Figure 4a, *p* < .05), whereas *COL3A1*, *TNC*, and *VEGF* were significantly increased compared with assay media controls (Figure 4a, *p* < .0001, *p* < .001, and *p* < .05, respectively). In HSAM CM‐treated tenocytes, gene expression of *TNC* and *VEGF* was significantly decreased compared with assay media (Figure 4a, *p* < .001, *p* < .05). There were no significant changes in gene expression for *ELN* or *DCN* in any groups.

Proteoglycan and collagen deposition are essential to providing a functional ECM during tendon repair (Voleti et al., 2012). After 1 week of treatment with dACM CM or HSAM CM, evaluation of tenocyte deposition of sGAGs showed trends for increased deposition with both treatment groups compared with assay media, but these results were not statistically significant (Figure 4b). When collagen deposition was evaluated, tenocytes treated with dACM CM, but not HSAM, resulted in significantly higher levels of soluble collagen than did assay media controls (*p* < .05, Figure 4c).

### 3.5 Tenocyte response to inflammatory cytokines

Although inflammation is a critical part of the healing process, it has been hypothesized that ineffective healing is often a result of a prolonged or excessive inflammatory phase (Thomopoulos, Parks, Rifkin, & Derwin, 2015) and that modulation of inflammation early on in the tendon repair process could result in improved outcomes (Hays et al., 2008). Therefore, we were interested in evaluating the overall response of tenocytes under inflammation and how that response changed with the addition of cytokines released from dACM or HSAM. For these studies, tenocytes were treated with either TNF‐α or IL‐1β for 96 hr. The rationale for using TNF‐α and IL‐1β in these experiments is due to their upregulation during the inflammatory response following tendon injury (Manning et al., 2014), and the concentrations used in these experiments were selected on the basis of prior studies (John et al., 2010; Tsuzaki et al., 2003). In control and TNF‐α treatment groups, tenocytes treated with dACM CM had significantly decreased expression levels of *TGFB1* than did assay media controls (*p* < .0001, Figure 5a). For HSAM groups at 1 ng/ml TNF‐α, releasate from HSAM resulted in significantly decreased expression levels of *TGFB1*. In addition to gene expression, protein concentration was confirmed using ELISAs to measure protein production and release from the groups. With the use of ELISAs, a significant reduction in production of TGF‐β1 over 96 hr using both HSAM CM and dACM CM was found (Figure 5b). In response to IL‐1β stimulation, there were no significant differences in gene expression between groups under inflammatory stimulus (Figure 5c, significantly reduced TGF‐β1 in control group); however, both HSAM CM and dACM CM resulted in a reduction in TGF‐β1 production in the 0.1 ng/ml IL‐1β group (Figure 5d). Interestingly, HSAM CM also resulted in significant reduction in TGF‐β1 production with 1 ng/ml IL‐1β (Figure 5d, *p* < .001). Although some TGF‐β1 is thought to be beneficial to tendon healing, a reduction in TGF‐β1 production could be beneficial as studies have suggested that high levels of TGF‐β1 promote scar and adhesion formation (Katzel et al., 2011). Additionally, a reduction of TGF‐β1 during tendon healing has been shown to improve the range of motion of healed tendons (Xia et al., 2010) through reduced adhesion formation.

MMPs are responsible for degradation and reconstruction of the ECM. MMP‐1 has a specific role in cleaving collagen fibers (Nagase, Visse, & Murphy, 2006) and plays an important role during wound healing, specifically during re‐epithelialization (Gill & Parks, 2008); however, overexpression of MMPs can lead to excessive breakdown of the ECM. Additionally, production and release of MMPs are known to be stimulated by inflammatory molecules (John et al., 2010; Tsuzaki et al., 2003). After 96 hr of treatment with dACM CM and TNF‐α (all concentrations), *MMP1* expression and protein production were significantly upregulated compared with assay media (Figure 5e,f). Conversely, following inflammatory stimulation, ELISAs showed significantly decreased levels of MMP‐1 production (with HSAM CM) than did assay media in both 1 and 0.1 ng/ml TNF‐α groups (Figure 5f, *p* < .05). For control conditions (without inflammation), stimulation with dACM releasate resulted in upregulation of *MMP1* and corresponding protein production (Figure 5e,f, *p* < .001). When stimulated with IL‐1β, neither dACM CM nor HSAM CM treatment groups resulted in any significant differences in MMP‐1 gene expression or production than did assay media controls (Figure 5g,h).

### 3.6 Tenocyte culture with dACM and HSAM

**Figure 6:**
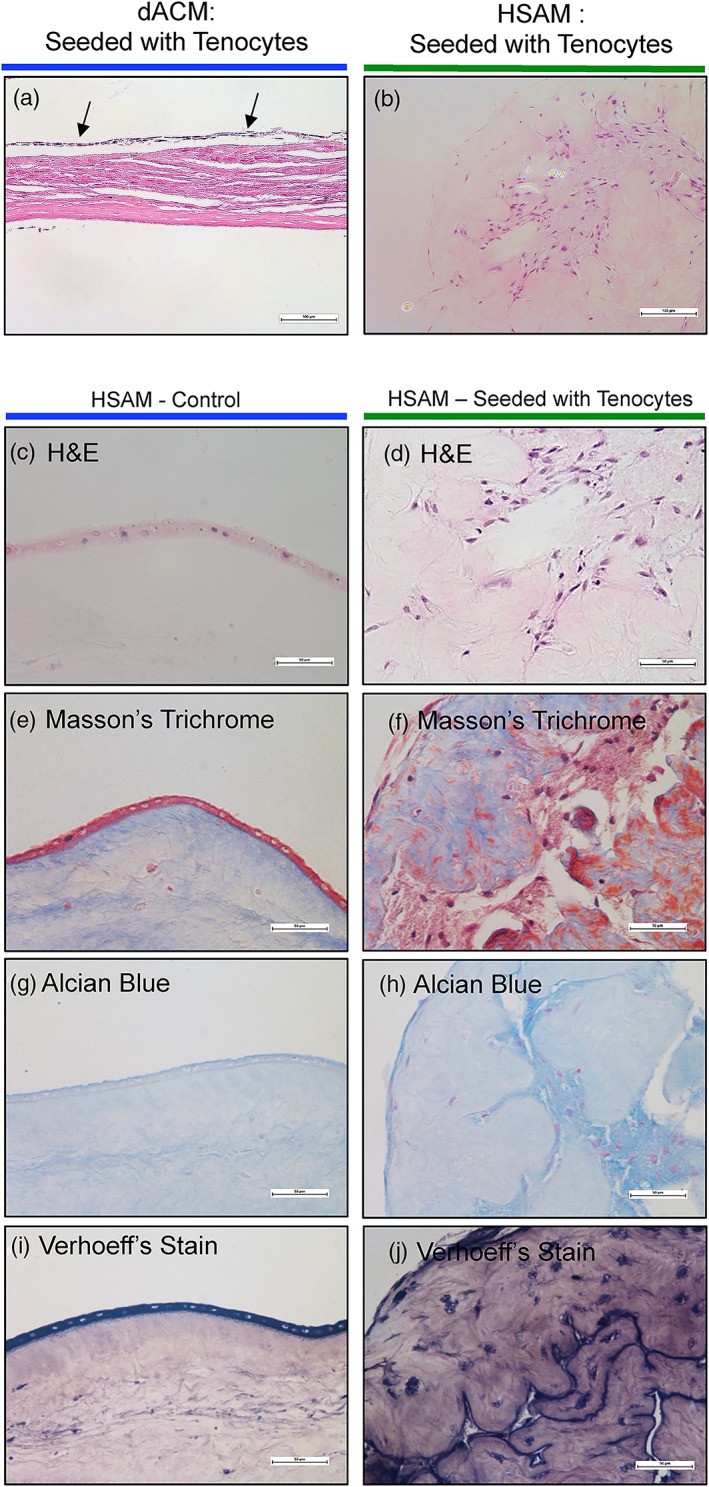
Evaluation of tenocyte interaction with dACM and HSAM. Representative hematoxylin and eosin (H&E) images of (a) dACM and (b) HSAM seeded with tenocytes. Scale bars indicate 100 μm. Results of HSAM seeded with or without tenocytes after 4 weeks. Representative images of (c,d) H&E, (e,f) Masson's trichrome, (g,h) Alcian Blue, and (i,j) Verhoeff's stain. Scale bars indicate 50 μm. dACM, dehydrated amnion/chorion membrane; HSAM, hypothermically stored amniotic membrane

In addition to levels of growth factors, and how those released factors interact with tenocytes, the interactions between tenocytes and the ECM of these grafts were evaluated. To do this, tenocytes were seeded onto either dACM or HSAM grafts for up to 4 weeks (Figure 6a,b). H&E staining revealed significant tenocyte attachment and migration into the HSAM graft (Figure 6b), whereas tenocytes cultured on dACM grafts attached but did not migrate into the graft (remained in a monolayer atop the graft, Figure 6a). To further evaluate how tenocytes interacted with HSAM grafts, a time course study was completed evaluating tenocytes seeded at 3, 7, 14, and 21 days on HSAM grafts (Figure S1). These results demonstrated how tenocytes were able to migrate into and remodel the HSAM graft over time. Additional staining at 28 days also revealed that tenocyte culture on HSAM resulted in increased collagen production as determined by Masson's trichrome staining (Figure 6e,f), increases in proteoglycan production as determined by Alcian Blue (Figure 6g,h), and elastin production as determined by Verhoeff's stain (Figure 6i,j). Additionally, remodeling of HSAM and deposition of molecules important for tendon repair (collagen I/III, tenomodulin, and decorin) were further evaluated using IHC staining (Figure S2). Images A, C, E, and G are control samples (HSAM with no tenocytes), whereas images B, D, F, and H are seeded samples (HSAM seeded with tenocytes). HSAM that was seeded with tenocytes qualitatively showed increased IHC staining for collagen III, tenomodulin, and decorin. We hypothesize the differences in tenocyte interaction between dACM and HSAM grafts is at least in part due to the preservation methods; hypothermic storage of the HSAM graft allows for maintenance and preservation of the ECM structure, which provides an open scaffold for the tenocytes to migrate through. In contrast, dehydration results in the removal of moisture, which in turn has been reported to result in a compressed ECM structure (McQuilling, Vines, Kimmerling, & Mowry, 2017). This dense structure and lack of pores, although structurally beneficial for adhesion barrier applications, likely prevents tenocytes from invading and migrating through the dACM graft.

## 4 DISCUSSION

In this work, we have studied two different grafts to evaluate whether differences in composition and processing would result in varying responses. A summary of high‐level study findings has been provided in Table 1. In this study, dACM and HSAM grafts were evaluated for total growth factor content and growth factor release from the membranes. In addition to verifying the presence and release of these factors, their ability to support key processes for tendon repair was evaluated, including proliferation and migration of tenocytes, expression and deposition of key ECM molecules, tenocyte responses to inflammatory signals, and the extent of tenocyte interactions with the ECM.

**Table 1:** Summary of results from dACM and HSAM grafts

Observed Response	dACM	HSAM
Composition	Significantly higher levels of bFGF and IGF‐I per cm^2^ compared with HSAM	Contains growth factors relevant to tendon repair
Release	Similar levels of growth factors released in vitro	
Proliferation	Increases of up to 3.5‐fold	Increases of up to 2.2‐fold
Migration (Boyden chamber)	Increases of up to 2.27‐fold	Increases of up to 6.02‐fold
Migration (scratch assay)	No changes observed compared with control	Significant increase in closure compared with control
ECM expression	Significant increases in collagen III, tenascin C, and VEGF expression	Significant decreases in collagen I, tenascin C, and VEGF expression
ECM production	Significant increases to soluble collagen production	No significant increases compared with controls
Response to TNF‐α and IL‐1β	Overall decreased TGF‐β1 production	Overall decreased TGF‐β1 production
	Overall increased MMP‐1 production	Overall decreased MMP‐1 production
Tenocyte interactions	Cell attachment, without infiltration	Cell attachment with cell infiltration and remodeling

Abbreviations: bFGF, basic fibroblast growth factor; dACM, dehydrated amnion/chorion membrane; ECM, extracellular matrix; HSAM, hypothermically stored amniotic membrane; IGF‐I, insulin‐like growth factor I; IL‐1β, interleukin 1 beta; MMP‐1, matrix metalloprotease‐1; TNF‐α, tumor necrosis factor alpha; VEGF, vascular endothelial growth factor.

Previous studies on tendon healing have demonstrated that several key growth factors are required to promote cellular proliferation and tissue repair at the injury site (Molloy et al., 2003; Nourissat et al., 2015). These include aFGF, bFGF, IGF‐I, PDGF‐BB, and TGF‐β1, all of which were evaluated in this study. Various studies have demonstrated that these growth factors, when provided individually following an injury, can expedite the repair process (Fukui, Katsuragawa, Sakai, Oda, & Nakamura, 1998; Kobayashi, Kurosaka, Yoshiya, & Mizuno, 1997; Kurtz, Loebig, Anderson, DeMeo, & Campbell, 1999) and potentially improve the quality of the outcome (Chan et al., 2000; Hildebrand et al., 1998; Kurtz et al., 1999; Letson & Dahners, 1994). Both dACM and HSAM grafts had physiologically relevant concentrations of all five growth factors evaluated, but dACM contained significantly more bFGF and IGF‐I. Interestingly, when further evaluating the release of growth factors over time by evaluating CM, these differences were no longer discernable for any growth factors measured. We hypothesize that this is due to the processing methodology differences; HSAM is a fresh, aseptically processed graft with minimal processing steps that is stored at hypothermic temperatures, whereas dACM has been dehydrated and terminally sterilized, resulting in significant perturbations to the ECM structure.

Following assessment of important factors in both grafts, the ability of these released growth factors to support tendon repair mechanisms was evaluated in vitro. We observed that growth factors released from both HSAM and dACM triggered an increase in tenocyte proliferation; however, dACM CM significantly increased tenocyte proliferation than did HSAM conditions. When comparing HSAM with dACM, the reasons for increases in proliferation are not obvious because there are no significant differences in the levels of mediators found in CM measured in this study (Figure 1b). Although not significant, the trends for higher bFGF in dACM may be responsible for this finding. It has been reported that the combined presence of IGF‐I, PDGF‐BB, and bFGF has a synergistic effect on tenocyte proliferation (Costa et al., 2006), pointing to the potential value of a multifaceted treatment modality. Alternatively, this observed difference may be due to increases in other factors found within dACM or HSAM, which were not evaluated in this study.

Although proliferation of tenocytes is important, migration of tenocytes to the injury site is also essential for repair. Therefore, the ability of each graft to facilitate and enhance tenocyte migration was evaluated. With the use of both Boyden chamber and scratch assays, it was demonstrated that releasate from HSAM promotes significantly greater tenocyte migration than dACM CM. We also evaluated the effect that ECM components might have on tenocytes by directly seeding cells onto dACM or HSAM grafts and evaluating subsequent adhesion, migration, and remodeling of the graft. We found that tenocytes seeded on dACM for up to 4 weeks lined up along the graft and did not migrate into the matrix; however, when tenocytes were seeded on HSAM grafts, they readily attached and infiltrated the matrix. Additionally, further staining revealed remodeling of the matrix by tenocytes with deposition of elastin, sGAGs, collagen III, tenomodulin, and decorin throughout HSAM grafts. For HSAM, similar results were seen by our group when evaluating fibroblast interactions with HSAM; after 2 weeks of culture, fibroblasts deposited fibronectin and collagen III throughout the graft (McQuilling, Vines, & Mowry, 2017). The results seen with tenocytes lining up along dACM are not indicative of migration and infiltration into the graft; however, these results are encouraging and supportive of clinical studies pointing to the value of dehydrated placental membranes as a surgical adhesion barrier (Subach & Copay, 2015).

Tenocytes cultured in CM from either dACM or HSAM exhibited blunted TGF‐β1 production following TNF‐α and IL‐1β stimulation, a finding that can be considered beneficial to the repair process as excessive TGF‐β1 is associated with scarring and adhesions (Chang, Thunder, Most, Longaker, & Lineaweaver, 2000; Farhat et al., 2015; Katzel et al., 2011; Xia et al., 2010). Previous work has demonstrated that too much TGF‐β1 can result in adhesion formation, and reduction of the available TGF‐β1 can improve the range of motion of the healed tendon (Xia et al., 2010).

In contrast, tenocytes cultured with TNF‐α and dACM CM resulted in a significant increase in MMP‐1 production, whereas culturing with HSAM CM resulted in a significant decrease in MMP‐1 production. Depending on the phase of healing, these results are somewhat contradictory. During the inflammatory and remodeling phases, expression of MMP‐1 is necessary to facilitate ECM breakdown; however, during other phases, overexpression may be harmful to the overall repair process. One limitation to this study is that differences in levels of protease inhibitors were not investigated; various placental‐derived tissue formulations have demonstrated in vitro inhibition of MMPs, collagenase, and elastase due to high levels of protease inhibitors (Kim, Kim, Na, Jeong, & Song, 2000; Litwiniuk et al., 2012).

When evaluating gene and ECM production in response to HSAM and dACM CM, several notable differences in responses were observed. Tenocyte culture in HSAM CM resulted in the downregulation of several ECM genes including *TNC* and *COL1A1*; however, when cultured in dACM CM, several ECM genes were upregulated including *TNC* and *COL3A1*. Additionally, deposition of collagen was unaltered compared with negative controls in HSAM CM groups but was significantly increased in tenocytes cultured with dACM CM. ECM deposition was qualitatively observed with tenocytes cultured on HSAM, but tenocytes remained lined on the surface of the dACM graft. Both dACM and HSAM have been previously reported to contain VEGF (Koob et al., 2013; McQuilling, Vines, & Mowry, 2017). In this study, we found that exposure of tenocytes to releasate from dACM resulted in significantly increased VEGF expression by tenocytes. In addition to the well‐known classical role of VEGF as a powerful stimulator of angiogenesis, a study by Zhang et al. found that the injection of exogenous VEGF to rat Achilles tendon repairs resulted in increased tensile strength at 1 and 2 weeks (Zhang et al., 2003).

One limitation of this work is the limited proteomic evaluation of these grafts. The analysis within this study consisted of five growth factors known to be relevant to tendon repair; however, these factors are only a fraction of the growth factors known to be present within placental tissues. Furthermore, in vitro work with tenocytes is limited by both the lack of complexity of cellular phenotypes found in vivo and the tendency for phenotypic drift in tenocyte cultures (Yao, Bestwick, Bestwick, Maffulli, & Aspden, 2006). Future in vitro work will focus on how these tissues may interact with other cells important to the repair process. Additional in vivo work evaluating these membranes and their effect on tendon repair is necessary. Recently, we have reported a reduction of tendon rerupture rates and trends for improved mechanical properties when Achilles tendon repair was modified with dACM in a diabetic rat model (McQuilling et al., 2019). Looking ahead, there may be a role for both dACM and HSAM grafts in supporting tendon repair depending on what pathways are clinically deficient.

## 5 CONCLUSION

This research is the first to evaluate multiple types of commercially available placental‐derived grafts as an adjunct to tendon repair. In sum, we found that both dACM and HSAM contain and release growth factors relevant to tendon repair. Interestingly, although both grafts promoted tendon repair mechanisms, there were clear differences in how dACM and HSAM affected tenocytes in vitro. These differences are interesting and may be a result of the layers included (amnion vs. amnion/chorion), processing technique (aseptic, hypothermic storage vs. terminally sterilized, dehydrated), or some combination of these factors. Although this study illustrates several potential ways in which placental‐derived membranes may support tendon repair, further in vivo work will be necessary to better understand these results.

## CONFLICT OF INTEREST

JPM, KAK, and KCM are paid employees of Organogenesis.

## FUNDING INFORMATION

This Study was funded by Organogenesis.

## Supporting information


**Table S1.** List of TaqMan Probes (ThermoFisher Scientific).

**Figure S1.** Time course evaluation of tenocyte interaction with HSAM. Representative hematoxylin and eosin (H&E) images of HSAM seeded without or with tenocytes at (A, B) 3 days, (C, D) 7 days, (E, F) 14 days, and (G, H) 21 days. Scale bars indicate 100μm.

**Figure S2.** Immunohistochemical (IHC) staining of tenocyte interaction with HSAM. Representative images of HSAM seeded without or with tenocytes of (A, B) Collagen I, (C, D) Collagen III, (E, F) Tenomodulin and (G, H) Decorin. Scale bars indicate 50μm.
