# Supplementary figures and images for: Evaluation of two distinct placental‐derived membranes and their effect on tenocyte responses in vitro
Source: J Tissue Eng Regen Med. 2019 Jun 13;13(8):1316–30. doi: 10.1002/term.2876 (PMC6771722; doi:10.1002/term.2876)

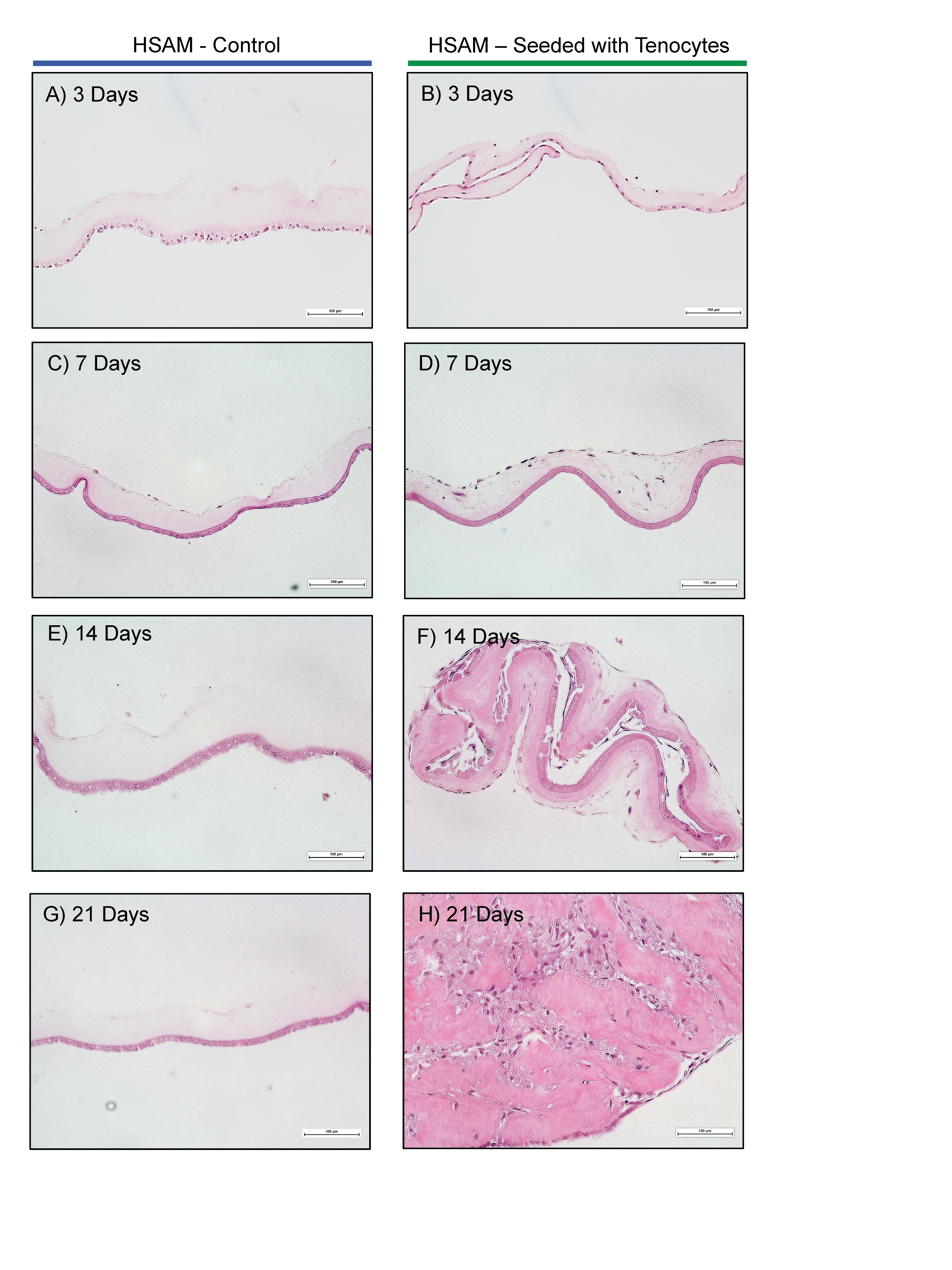

Supplement: Supplementary file 2 — Figure S1. Time course evaluation of tenocyte interaction with HSAM. Representative hematoxylin and eosin (H&E) images of HSAM seeded without or with tenocytes at (A, B) 3 days, (C, D) 7 days, (E, F) 14 days, and (G, H) 21 days. Scale bars indicate 100μm. [file TERM-13-1316-s001.tiff]

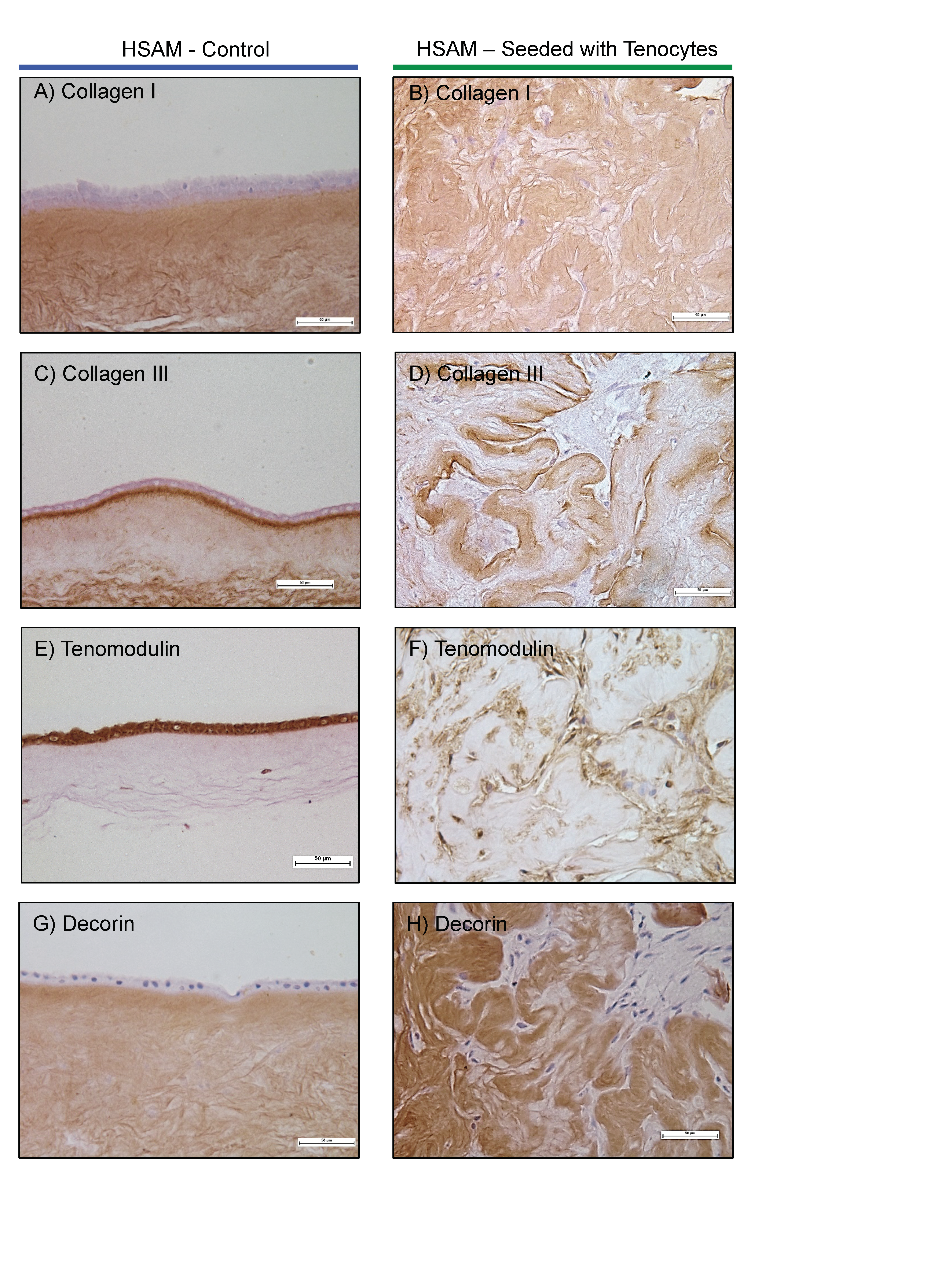

Supplement: Supplementary file 3 — Figure S2. Immunohistochemical (IHC) staining of tenocyte interaction with HSAM. Representative images of HSAM seeded without or with tenocytes of (A, B) Collagen I, (C, D) Collagen III, (E, F) Tenomodulin and (G, H) Decorin. Scale bars indicate 50μm. [file TERM-13-1316-s002.tiff]
